# Identification of Gold Sensing Peptide by Integrative Proteomics and a Bacterial Two-Component System

**DOI:** 10.3389/fchem.2017.00127

**Published:** 2017-12-22

**Authors:** I-Son Ng, You-Jin Yu, Ying-Chen Yi, Shih-I Tan, Bo-Chuan Huang, Yin-Lung Han

**Affiliations:** ^1^Department of Chemical Engineering, National Cheng Kung University, Tainan, Taiwan; ^2^Green Energy and Environment Research Laboratories, Natural Resources, Technology Division, Industrial Technology Research Institute, Hsinchu, Taiwan

**Keywords:** gold sensing peptide, *Tepidimonas fonticaldi*, proteomics, two-component system, PmrAB

## Abstract

The proteomics strategy was utilized to analyze and identify the gold adsorption proteins from *Tepidimonas fonticaldi* AT-A2, due to its outstanding performance in gold-binding and recovery. The results showed that three small proteins, including histidine biosynthesis protein (HisIE), iron donor protein (CyaY) and hypothetical protein_65aa, have a higher ability to adsorb gold ions because of the negatively charged domains or metal binding sites. On the other hand, the *Salmonella* PmrA/PmrB two-component system first replaces the iron (III)-binding motif using the peptide sequence from hypothetical protein_65aa, and this is then used to reveal the sensing and responsiveness to gold metal ions, which is totally different from the performance of traditional gold binding peptide (GBP) on the crystals on the surface of gold (111). We have successfully demonstrated an integrative proteomics and bacterial two-component system to explore the novel GBP. Finally, the heterologous over-expression of GBP by *E. coli* and the equilibrium of binding capacity for Au(III) have been conducted.

## Introduction

The chemical and physical techniques used in the recovery of precious metals (i.e., gold, platinum, silver, and copper) always require energy, as well as abundant chemical inputs or high-cost facilities, while also producing secondary wastes. Compared with chemical and physical approaches, bio-processes such as bio-adsorption, biodegradation, and bioremediation, based on the different kinds of microorganisms, can reduce both energy consumption and pollution, thus offering an eco-friendly, sustainable and multidisciplinary solution to this problem (Klaus-Joerger et al., 2001; Deplanche and Macaskie, 2008). In previous studies, a new strain *Tepidimonas fonticaldi* sp. nov. AT-A2 isolated from hot spring water (Chen et al., 2013), showed outstanding performance in gold adsorption, with significant specificity (Han et al., 2017).

Marc Wilkins established proteomics technology, which has been widely used since 1995 (Wilkins et al., 1995), becoming a powerful tool due to its comprehensive analysis of the identified proteins (Ling et al., 2015), modifications of proteins (Mann and Jensen, 2003), and even analysis of the metabolic fluxes occurring within cells (Ye et al., 2014; Ng et al., 2016). On the other hand, proteomics approach can be used to clarify and explore the metal-tolerant protein under different stress in plant (Chen et al., 2015). It is thus of interest to identify gold adsorption proteins using proteomics.

Prokaryote microorganisms usually respond to external stress by using a two-component regulatory system when the environment changes. In more detail, self-phosphorylation occurs when histidine protein kinase (HK), which is located on the cell membrane, detects a stimulus in the environment, and then a responsive regulator protein (RR) is activated after the phosphate group transfer HK to RR (Stock et al., 2000). A previous study found that the *Salmonella enterica* genus has a PmrA/PmrB two-component system to resist cytoplasmic trivalent iron ions or polymyxin B (Chen and Groisman, 2013). PmrB, as a transmembrane protein, has two pairs of specific amino acid sequences “EXXE” which can act as a sensor on the cell surface because self-phosphorylation occurs when sensing foreign ferric ions, and this then transfers the phosphate group to PmrA sequentially (Wösten et al., 2000). PmrA is active after phosphorylation and recognizes a promoter located in front of the PmrC sequence to trigger the downstream signaling gene. The cell can thus grow normally due to this regulation or inhibition of the relevant genes and specific proteins, and so reach a resistant response (Chen and Groisman, 2013).

The successful replacement of iron(III)-binding motif to sense Lanthanide ions occurred in a metal response system constructed by a PmrA/PmrB two-component protein through the signal of green fluorescent protein (GFP) have been successfully demonstrated by He's group (Liang et al., 2013). However, the GFP signal is not sensitive as the super-fold GFP (sfGFP). In this study, we aim to analyse and identify the gold adsorption proteins from *T. fonticaldi* sp. nov. AT-A2, which has outstanding gold adsorption capability, by integrative proteomics and a bacterial two-component system with a higher fluorescence signal.

## Materials and methods

### Separation of Au binding protein/peptide by ultra-filtration

We utilize 30 kDa, 3 kDa ultrafilter and His-trap affinity chromatography to separate the protein mixture. We then analyze the gold adsorption ability using SDS-PAGE electrophoresis, and identify the predicted proteins with Q-TOF-MS. We measure the metal concentration using ICP-OES (HORIBA ULTIMA 2000, Japan) and detect the fluorescence intensity with a fluorescence spectrophotometer (Molecular Devices SpectraMax M2, USA). The excitation and emission wavelengths for detection of the green fluorescence intensity were set at 485 nm and 510 nm, respectively.

### Metal adsorption experiments and ICP-OES analysis

The 0.32 mL of fresh microbial cells was mixed with 1.28 mL Au^3+^ metal ions solution in a 2-mL tube, to obtain final concentrations of metal ions at 1 ppm to 80 ppm and a biomass as 0.25 g/L. The suspension was shaken at 70 rpm for 30 min at room temperature, and then centrifuged at 12,000 × g for 5 min to separate the cells and supernatant. The supernatants were filtered using a 0.22 μm membrane followed by ICP-OES (HORIBA ULTIMA 2000, Japan) analysis to determine the un-adsorbed metal concentration.

### Proteomics analysis of gold adsorption proteins

The proteins after ultra-filtration by 30 kDa cut-off were subjected to electrophoresis for further separation followed by MALDI-TOF-TOF analysis using an ABI QSTAR Pulsar i-System (Applied Biosystems, USA). Raw spectral data were further processed using the Data Explorer 4.6 software (Applied Biosystems, USA). The peak list files were used to query the NCBI database using the Mascot program.

### Construction of the two-component system

The strains, plasmids and primers used in this study are shown in Table 1. *BasS* gene in the *E. coli* MG1655 chromosome should be knock-out to obtain the MG1655ΔBasS::Kan for the two-component sensing system. The vector pKD46, primers of BasS-HR-L-Kan and Kan-R-HR-BasS were used for gene knock-out of BasS in relation to the procedure of lambda-red inactivation of chromosomal genes in *E. coli* (Datsenko and Wanner, 2000). Then, the pMD19T-P_BAD_-BasRS and pSB1C3-BSpmrC(S)-sfGFP were constructed in DH5α. First, the genome DNA of E. coli MG1655 was extracted and used for amplifying BasRS by primers of BasR/S-*Kpn*I-F and BasR/S-*Sal*I-R. The plasmid backbone, pMD19T-P_BAD_ was amplified from our lab stock, pMD19T-P_BAD_-lysis, by primers of pBAD-*Sal*I-F and pBAD-*Kpn*I-R. The vector and insert gene were digested with *Sal*I/*Kpn*I. The same procedure and primers of BSpmrC-*Eco*RI-F and BSpmrC-*Bam*HI-R were used for construction of pSB1C3-BSpmrC(S)-sfGFP, for which the vector came from pSB1C3-sfGFP and the insert [i.e., BSpmrC(S)] was synthesized using IDT integrated DNA technology (USA). The primers of Au-F and Au-R were used to replace the original sequence of the Fe binding site in pMD19T-P_BAD_-BasRS and thus pMD19T-P_BAD_-BasRS-65aa was obtained, while the primers of AraC-Amp-F and Amp-R were applied to the insert araC from pKD46, and this was then integrated into pMD19T-P_BAD_-BasRS to obtain pBAD-BasRS-65aa. All the positive constructions in Table S1 were further confirmed after colony PCR of the target gene, plasmid digestion and sequencing.

**Table 1:** Strains, plasmids, and primers used in this study.

**Material**	**Genotype or description**	**Sources**
**STRAINS**		
*Tepidimonas fonticaldi* sp. nov. AT-A2	Wide type	ITRI
*E. coli* DH5α	4,507,030 bp, F^−^*endA1 glnV44 thi-1 recA1 relA1 gyrA96 deoR nupG purB20* φ80d*lacZ*ΔM15 Δ(*lacZYA-argF*)U169, hsdR17(*r_*K*_*^−^*m_*K*_*^+^), λ^−^	Lab stock
*E. coli* MG1655	4,646,332 bp, F^−^λ^−^ rph-1 INV(rrnD, rrnE)	Lab stock
*E. coli* BL21(DE3)	*E. coli* str. B F^−^*ompT gal dcm lon hsdS_*B*_*(*r_*B*_*^−^*m_*B*_*^−^) λ(DE3 [*lacI lacUV5-T7p07 ind1 sam7 nin5*]) [m*alB*^+^]_K−12_(λ^S^)	Lab stock
**PLASMIDS**		
pKD46	6,329 bp, Amp^R^ P_araB_ promoter, repA101ts, lambda Red, tL3, araC	Prof. Yun-Peng Chao
pMD19T-P_BAD_-lysis	3,178, Amp^R^, P_BAD_ promoter	Lab stock
pSB1C3-sfGFP	3,099 bp, Cm^R^, pUC ori, P_lacI_ promoter, B0034	Lab stock
pET28a(+)	5,369 bp, Kan^R^ T7 *lac* promoter, His∙Tag and T7∙Tag	Lab stock
**Primers**	**Sequences (5′ → 3′)**	
BasS-HR-L-Kan	TAACTACCGT GTTCAGCGTG CTGGTGGTCA GCAGCTTTCTTTAGAAAAAC TCATCGAGCA	This study
Kan-R-HR-BasS	CTATATGCTG GTCGCGAATG AGGAAAACTA ATTGAATCTGTTTCTACGGG GTCTGACGCT	This study
BasR/S-*Kpn*I-F	CCGGTACCATGAAAATTCTGATTGTTGAAGACGAT	This study
BasR/S-*Sal*I-R	TAGTCGACTTATATCTGGTTTGCCACGTACTGATC	This study
pBAD-*Sal*I-F	GCGTCGACTACTAGAGCCAGGCATCAAATAAAAC	This study
pBAD-*Kpn*I-R	GCGGTACCCTCTAGTATTTCTCCTCTTTCTCTAG	This study
BSpmrC-*Eco*RI-F	CCGAATTCTTACACTGGTGCCATATCTTTACACCT	This study
BSpmrC-*Cla*I-R	ATATCGATCACGGTGTTTCCATCGAACAAAGTGCG	This study
BSpmrC-*Bam*HI-R	GTGGATCCGTTGATGCGTCCATCGATTCG	This study
Au-F	TGAAAGCGATTACCCAGGCGATTCGCGCGCTGGA TCCGCAGGCGGTCGCCAGCCTGATTG	Insert GBP from 65aa
Au-R	CGCAATGGCCGCAGCTCATGCCATCCACGGTAAA CACATGCTGTAGCCAGAAGACGCTGA	Insert GBP from 65aa
AraC-Amp-F	GTCCACATTGATTATTTGCACGGCGAATTCGC	Amplify araC from pKD46
Amp-R	TGCCTCACTGATTAAGCATTGGTAA	Amplify araC from pKD46
65aa-*Eco*RI-F	CAGAATTCATGCAGCATGTGTTTACCGTG	Amplify 65aa
65aa-*Xho*I-R	TACTCGAGATCGCGCACGGTATAGCCTTC	Amplify 65aa

*Kan^R^, Kanamycin-resistance; Amp^R^, Ampicillin-resistance. EcoRI (GAATTC), XhoI (CTCGAG), NcoI (CCATGG), NdeI (CATATG), EcoRV (GATATC), KpnI (GGTACC), SalI (GTCGAC), ClaI (ATCGAT), BamHI (GGATCC) restriction sites are underlined*.

### Measurement of the fluorescence intensity

The precipitated cells were resuspended in deionized water to a final density, with as 0.5 g/L and 200 μL was added into a 96-well plate. The fluorescence intensity was determined using a fluorescence spectrophotometer (Molecular Devices SpectraMax M2, USA). The excitation and emission wavelengths for detection of the green fluorescence intensity were set at 485 and 510 nm, respectively. All experiments were conducted three times.

### Cloning, recombinant expression, and purification of 65aa in *E. coli*

The DNA and amino acid sequence of 65aa peptide are shown in Table S2. The target gene is obtained via Integrated DNA Technologis Company (IDT) in USA. The 65aa peptide was amplified by primers of 65aa-*Eco*RI-F and 65aa-*Xho*I-R and inserted between the T7 promoter and terminator of expression vector pET28a(+) and introduced into *E. coli* BL21(DE3). Recombinant colonies grown on LB-Kan plates were checked by colony PCR and double digestion by restriction enzymes *Eco*RI and *Xho*I. The cells harboring of pET28a-65aa plasmid were cultured in 100 ml of LB medium supplemented with 50 μg/ml kanamycin on a rotary shaker (200 rpm) at 37°C. When the cell density reached to OD_600_ at 0.6, it was induced by final concentration of 0.1 mM IPTG and further cultured for 8 h. The cells were washed three times and collected by centrifugation at 10,000 × g 4°C for 10 min. The cell suspension were adjusted to OD_600_ = 10 and then disrupted by a homogenizer at 30 kpsi (Constant system, OneShot Model, UK). The supernatant was centrifugal once again at 15,000 × g 4°C for 15 min and filtrated through a 0.22 μm membrane. A His-Trap affinity chromatography column was applied in GE Healthcare ÄKTA FPLC chromatography system. Fractions containing recombinant 65aa were eluted with 500 mM imidazole dissolving in sodium phosphate buffer (10 mM, pH 7.4). Finally, excess imidazole in the purified 65aa was removed by ultrafiltration with 3 kDa cut-off.

### Protein expression determined by SDS-PAGE

The gel was prepared with 0.1% SDS in 10% separating gel and 4% stacking gel. Tris-glycine buffer (pH 8.3) containing 0.1% SDS was used as the electrode buffer. The samples of 10 μl with ~0.1 mg protein were treated with the protein buffer and heated at 95°C for 5 min prior to apply in the gel. The proteins were visualized by staining with Coomassie blue R-250 and scanned on the Image scanner.

### Binding capacity of 65aa

The aqueous Au (III) solution varied from 0 to 1,200 mg/L and added into 50 mg/L protein suspension (i.e., purified 65aa) at a volume ratio of 1:1 and to get the final Au(III) at 0 to 600 mg/L and protein at 25 mg/L, respectively. The experiments of binding capacity were carried out in 15 ml tubes and incubated at room temperature for 1 h with shaker 70 rpm. The residual concentration of Au (III) was measured as the same procedure in aforementioned ICP-OES analysis. The binding affinity (*K*_*d*_) and *B*_*max*_ are obtained by fitting the data to the model of specific binding with Hill slope as following equation:

$$\begin{array}{l} B=Bmax\frac{x^{n}}{K_{d}^{n}+x^{n}} \end{array}$$

where *B*_*max*_ is the maximum binding ratio, *K*_*d*_ is the binding affinity and *n* is the equilibrium constant.

## Results and discussion

The gold adsorption proteins from strain AT-A2 were analyzed and identified using the proteomics strategy. Ultrafiltration and His-trap affinity chromatography were used to separate the protein mixture, and to detect the gold adsorption ability. As shown in Table 2, the 3–30 kDa fractions showed the highest ratio of gold adsorption, i.e., 1.75 mg Au on per mg protein, while the adsorption rate of the initial mixed proteins in supernatant and the proteins with a molecular weight larger than 30 kDa were 0.82 and 0.36, respectively. The protein fractions from 3 to 30 kDa were separated by SDS-PAGE electrophoresis, and then subjected to tandem MS/MS analysis. The results of Mascot protein identifications for three protein fractions [i.e., histidine biosynthesis protein (HisIE), iron donor protein (CyaY) and hypothetical protein_65aa (65aa)] in AT-A2 are summarized in Table 3. According to the predicted structure of proteins by SWISS-MODEL (https://swissmodel.expasy.org/), it was found that HisIE included domain with positive charge ion, while CyaY owned an alpha helix would stabilize the binding energy of metal, and hypothetical protein_65aa was supposed to be metal binding proteins. In summary, the gold binding ability was attributed to the negatively charged domain, alpha helix, or specific metal binding motif, such as sequence of MSCXXC. Compared to the gold binding peptide (GBP) (mhgktqatsgtiqs) discovered by Brown (Brown, 1997), which can detect gold-binding behavior on the structure of Au(111) (Brown et al., 2000), this novel and specific peptide from AT-A2 is used for detection of gold ions in the solution.

**Table 2:** Au adsorption ratio on proteins from different portions of *Tepidimonas fonticaldi* sp. nov. AT-A2.

**Sample**	**Protein conc. (ppm)**	Au^*^ adsorption (ppm)	**Adsorption ratio of Au/protein**
Supernatant	7.6	6.2	0.82
>30 kDa	25.0	8.9	0.36
3–30 kDa	23.6	41.2	1.75

The original Au^*^ is 51.7 ppm.

**Table 3:** Mascot protein identifications of fractional proteins <30 KDa in *Tepidimonas fonticaldi* sp. nov. AT-A2.

**No**.	**Protein name**	**Accession no**.	**Score**	**Mw (Da)**	**pI**	**Peptide match**
1	Histidine biosynthesis protein (HisIE)	WP_043698885	253	11,217	9.98	16
2	Iron donor protein (CyaY)	WP_043703651	181	12,257	5.06	19
3	Hypothetical protein_65aa	WP_058616432	28	7,299	5.16	5

**Figure 1:**
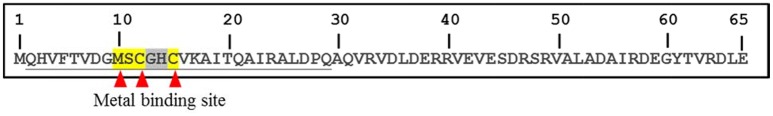
The metal binding site prediction in 65aa peptide by BLAST.

As shown in Figure 1, the predicted metal binding site in 65aa peptide was analyzed using the Basic Local Alignment Search Tool (BLAST). The MSCGHC site is located at the tenth to fifteenth amino acids in the sequence of 65aa. Therefore, we attempt to establish a responsive system to detect gold ions based on the PmrA/PmrB two-component system.

**Figure 2:**
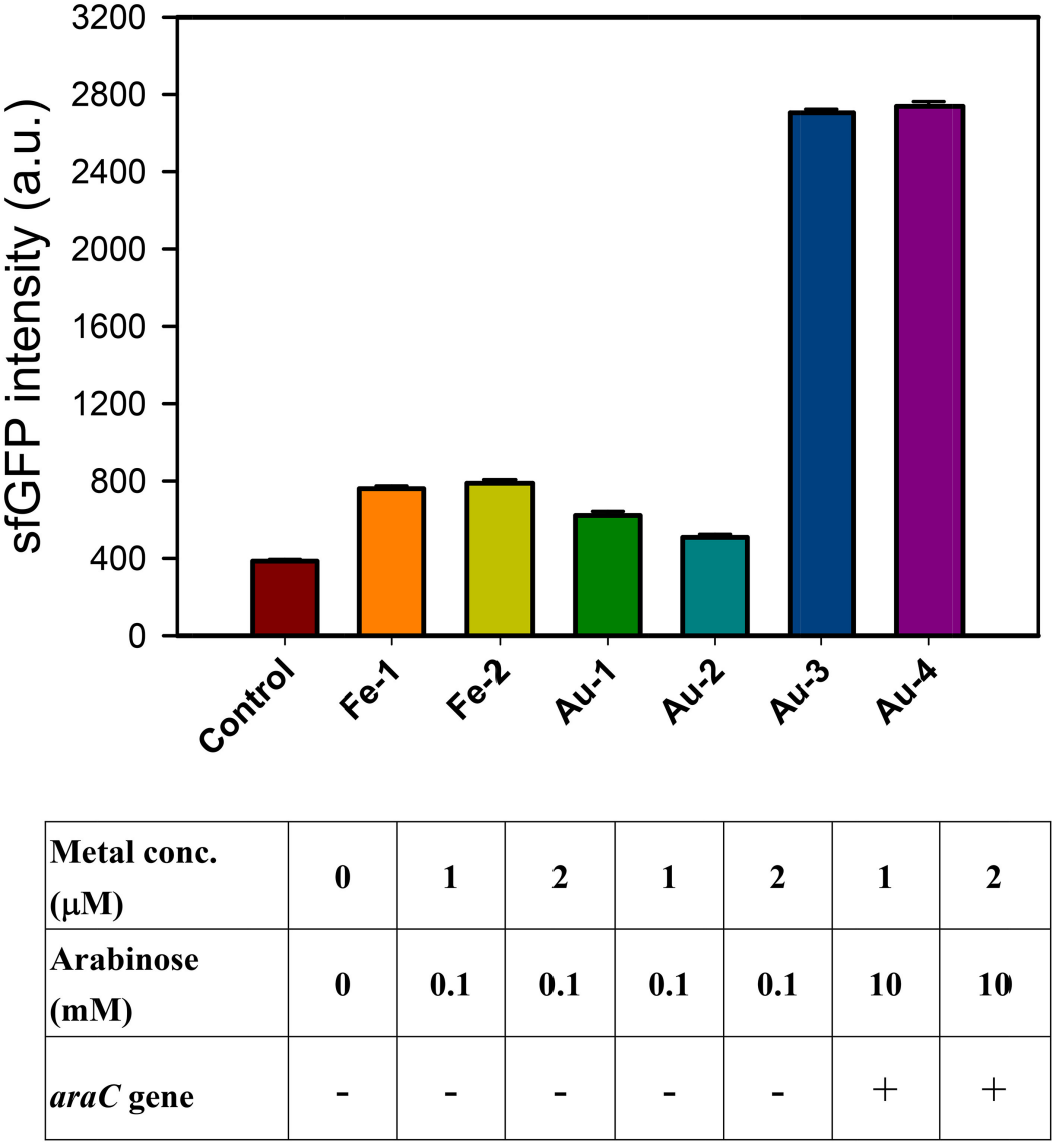
Engineered PmrA/PmrB-Au65/PmrC-sfGFP which sensing iron ion (Fe^3+^) and gold ion (Au^3+^). The sfGFP fluorescence response to different concentration of metal ions and arabinose concentration under araC gene regulation.

**Figure 3:**
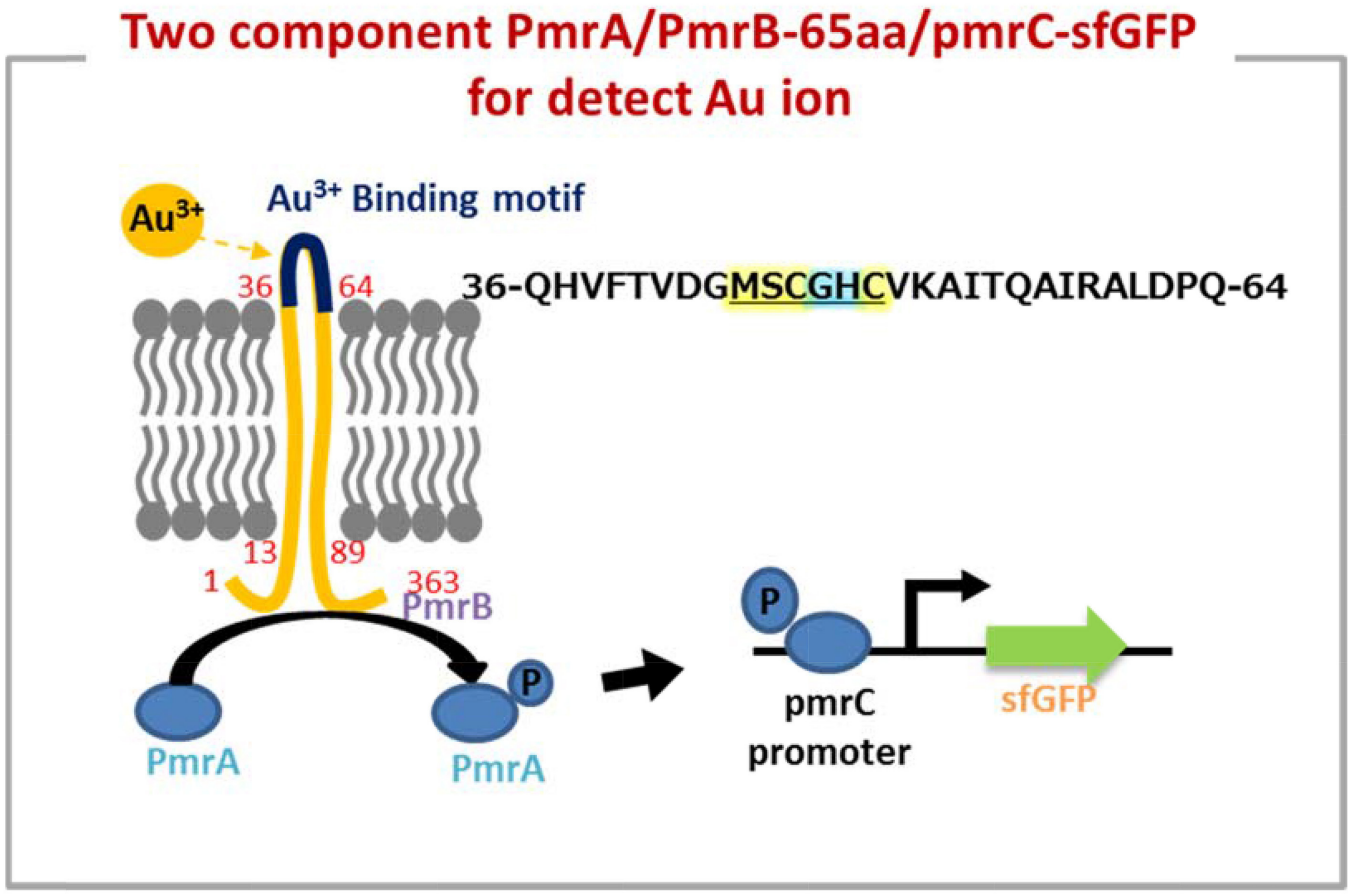
The two component system works on detection of gold ion by a novel peptide (Au-65) which explored by proteomics approach from *Tepidimonas fonticaldi* AT-A2. Design and construction of gold-responsive system based on PmrA/PmrB has successfully replaced amino acids of iron-binding motif (amino acid 36–64) from PmrB with QHVFTVDGMSCGHCVKAITQAIRALDPQ. The underlined is metal binding site prediction in 65aa.

The iron (III)-binding motif of *Salmonella* PmrA/PmrB two-component system was first replaced by the lanthanide-binding peptide sequence (Liang et al., 2013). In the resulting PmrA/PmrB/pmrC-GFP, the sensing intensity of GFP is around 100 a.u. at 100 μM Fe^3+^. We improved the sensitivity by using sfGFP, for which the vector construction included pSB1C3-BSpmrC(S)-sfGFP, pMD19T-P_BAD_-BasRS, pMD19T-pBAD-BasRS-65aa and pBAD-BasRS-65aa (Figure S1), with the confirmation shown in Figure S2. As a result, the sensitivity with regard to iron detection is 730 a.u. for 1 μM Fe^3+^ and 750 a.u. for 2 μM Fe^3+^ (Figure 2). When the amino acids of the iron-binding motif (amino acid 36–64) in PmrB is replaced with 36-QHVFTVDGMSCGHCVKAITQAIRALDPQ-64 from 65aa (Figure 3), the signals of sfGFP were 622 a.u. for 1 μM Au^3+^and 509 a.u. for 2 μM Au^3+^, respectively. Moreover, the sfGFP intensity increased to 2,705 a.u. for 1 μM Au^3+^and 2,738 a.u. for 2 μM Au^3+^ when the *araC* gene was strictly regulated under 10 mM arabinose (Figure 2).

In fact, the metal protein is usually a short metal-binding residue of only about 70–80 amino acids, which has a highly homologous conserved metal binding sequence of “MX1CX2X3C” (X stands for any amino acid). X1 is usually an amino acid of H, T, D, or S, while X2 and X3 are the small and hydrophobic or polar uncharged residues, such as A, G, or S (Shoshan and Tshuva, 2011). The minor differences in protein binding sites allow significant changes in the binding structure of the entire protein, and thus to sensing a particular metal (Opella et al., 2002). On the other hand, it has been reported that cysteine in the “CXXCGC” sequence region for metal-binding sites has a high affinity for nickel ions (Chan Chung et al., 2008). X-ray absorption spectroscopy experiments showed that the chemical binding of gold ion on egg shell biofilm was not only a simple electrostatic interaction, but also involved a complex ligand reaction (Ishikawa et al., 2002). Therefore, it is not easy to use physical chemistry to define the absorption of protein peptide. As the mechanism of PmrA/PmrB-65aa/pmrC-sfGFP shown in Figure 3, we validated a two-component system for the specific gold adsorbed peptide from AT-A2.

**Figure 4:**
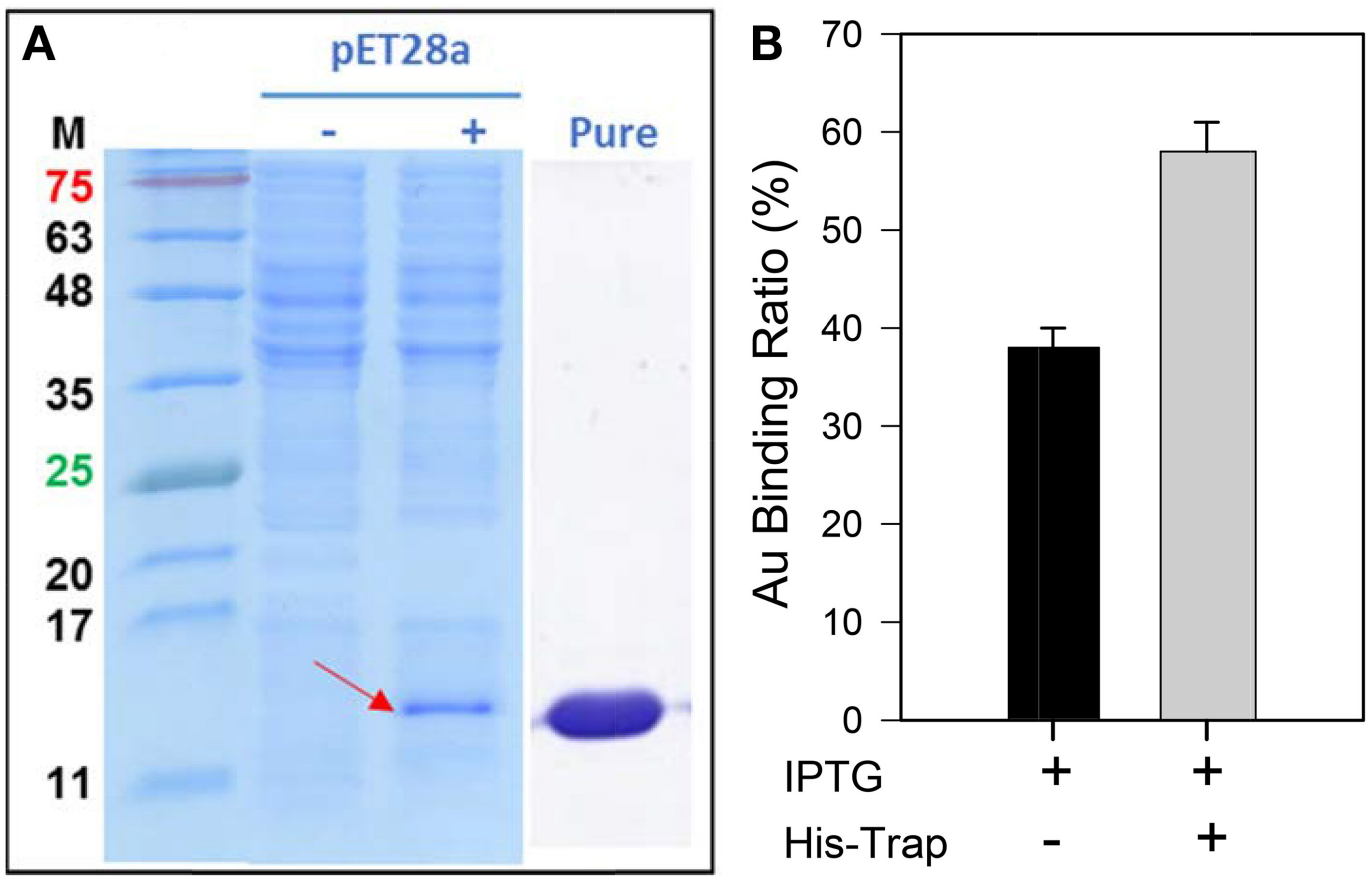
**(A)** The SDS-PAGE analysis of recombinant 65aa expression in *E. coli*. M means protein marker in molecular weight. The pET28a expression is under IPTG induction which (–) no IPTG and (+) with IPTG. **(B)** Gold binding ratio of 65aa with and without His-Trap affinity chromatography.

**Figure 5:**
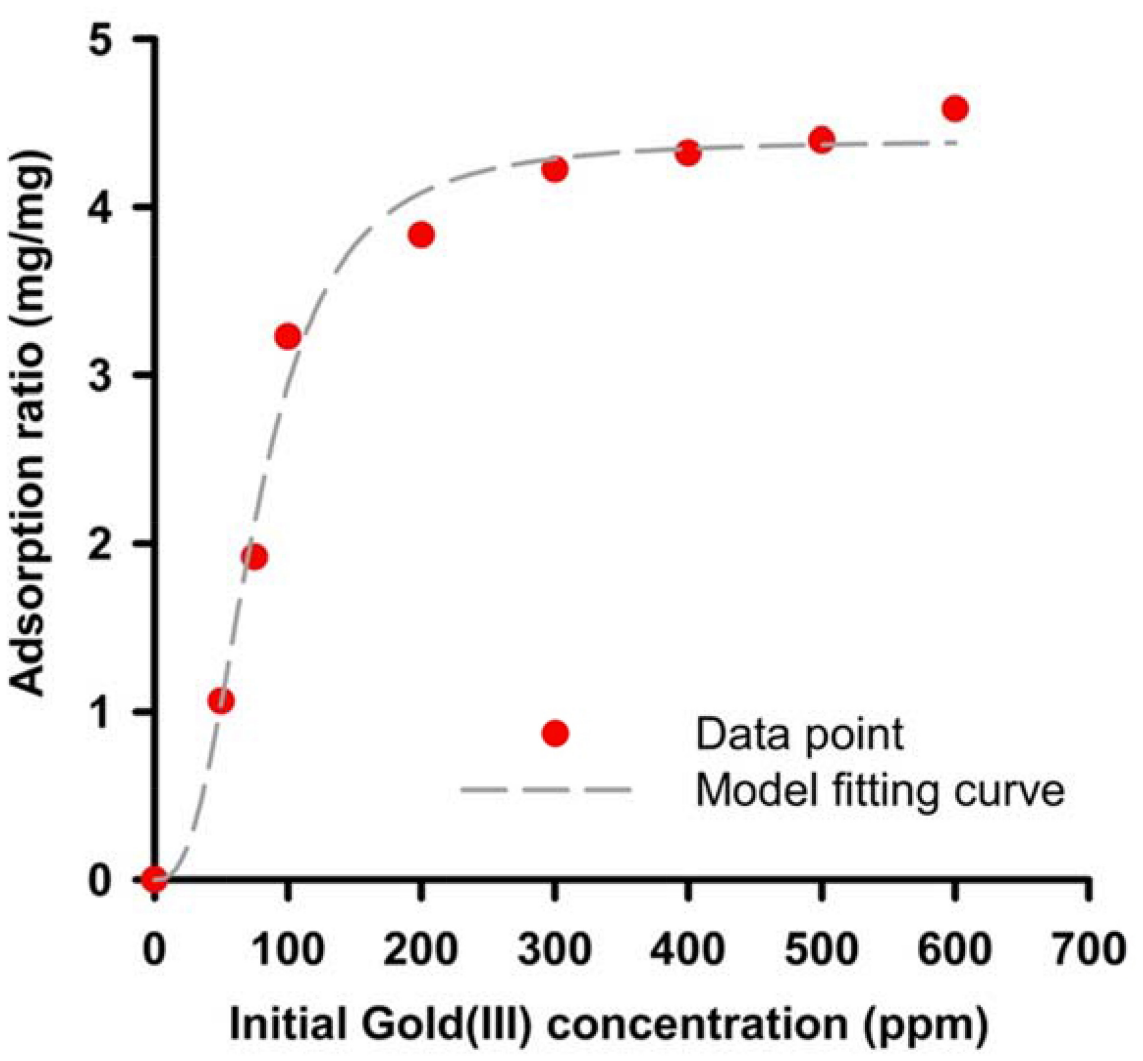
Binding capacity of recombinant 65aa at room temperatures. The adsorption data has shown in dot and model fitting is used by Hill function as *B* = *B*_max_^*^X^*n*^*/(*$K_{d}^{n}+$*X*^*n*^).

The GBP, 65aa, has further cloned to pET28a plasmid (Figure S3) and was heterologously expressed in *E. coli*. As shown in Figure 4A, the peptide of 65aa has been successfully expressed under IPTG induction and purified. The gold binding ratio is 38 and 58% for the crude extract protein and purified protein, respectively (Figure 4B). Further analysis of the binding capacity is shown in Figure 5. The equation of the fitting curve is followed by Hill function as below:

$$\begin{array}{l} B=4.4\;\left(\frac{mg}{mg}\right)\;\times \;\frac{x^{2.69}}{77.13\;{\left(ppm\right)}^{2.69}+x^{2.69}}\;\left({\text{R}}^{2}=0.989\right) \end{array}$$

Where *B* is binding ratio of adsorbed Au(III) per purified 65 a.a peptide and *X* is the initial Au(III) concentration. We can find out the maximal binding ratio (i.e., *B*_*max*_) is approximately at 4.4 (mg/mg), binding affinity *K*_*d*_ is 77.13 (ppm) and equilibrium constant *n* is 2.69. The results are close to the protein mixtures from the original strain AT-A2 (Han et al., 2017). However, this 65aa can be easily expressed in *E. coli* up to 200 mg/L, which the protein concentration is much higher than that from AT-A2 in 15 mg/L.

Finally, the selectivity of the GBP 65aa for different metal ions was tested in the printed circuit boards (PCBs) wastewater. As the result in Table 4, the gold adsorption was 86.8% which is higher than other elements, indicating that 65aa has high selectivity for Au(III) than Ag(I) and Cu(II).

**Table 4:** Comparison of gold binding peptide 65aa and AT-A2 in recovery of gold, silver, copper in the printed circuit boards (PCBs) wastewater.

**Elements**	**65aa**			AT-A2^*^		
	**Initial conc. (mg/L)**	**Residual conc. (mg/L)**	**Removal efficient (%)**	**Initial conc. (mg/L)**	**Residual conc. (mg/L)**	**Removal efficiency (%)**
Au^3+^	45	5.94	86.8	15	4.3	71.3
Ag^+^	0.72	0.70	0.03	<1	ND	ND
Cu^2+^	0.41	0.41	0	0.6	ND	ND
K^+^	142.2	123.90	12.9	309.6	302.3	2.4

* *The data is adapted from Han et al. (2017). ND, not determined*.

## Conclusion

The small proteins or peptides of the thermophilic strain *T. fonticaldi* AT-A2 are more likely to adsorb gold ions. The proteomics approach applied in this work contributed to exploring the gold binding proteins or peptides more effectively. The suspected proteins are in the negatively charged domain, and have alpha helix or a specific metal binding motif, such as MSCXXC. This is the first time that a PmrA/PmrB two-component responsive system has been used to confirm the gold adsorption proteins or peptides as sequence of QHVFTVDGMSCGHCVKAITQAIRALDPQ. Recombinant expression of 65aa in *E. coli* showed the binding affinity (*K*_*d*_) and maximum binding ratio (*B*_*max*_) was 77.13 ppm and 4.4 mg-Au(III)/mg-protein, respectively. This integration of proteomics and a bacterial two-component system provides an attractive approach to find novel functional proteins, with few limitations.

## Author contributions

I-SN: designed the research, did data analysis and wrote the manuscript; Y-JY: performed the proteomics experiment and initiated the two-component system; S-IT, Y-CY, and B-CH: accomplished the two-component system and did data analysis; Y-LH: provided comments and was involved in discussions.

### Conflict of interest statement

The authors declare that the research was conducted in the absence of any commercial or financial relationships that could be construed as a potential conflict of interest.
