# Supplementary material for: Identification of Gold Sensing Peptide by Integrative Proteomics and a Bacterial Two-Component System
Source: Front Chem. 2017 Dec 22;5:127. doi: 10.3389/fchem.2017.00127 (PMC5744191; doi:10.3389/fchem.2017.00127)
Supplement: Supplementary file 1 [file DataSheet1.pdf]

Supporting information

Identification of gold sensing peptide by integrative proteomics and bacterial two-component system

I-Son Ng[1*], You-Jin Yu[1], Ying-Chen Yi[1], Shih-I Tan[1], Bo-Chuan Huang[1], Yin-Lung Han[2]

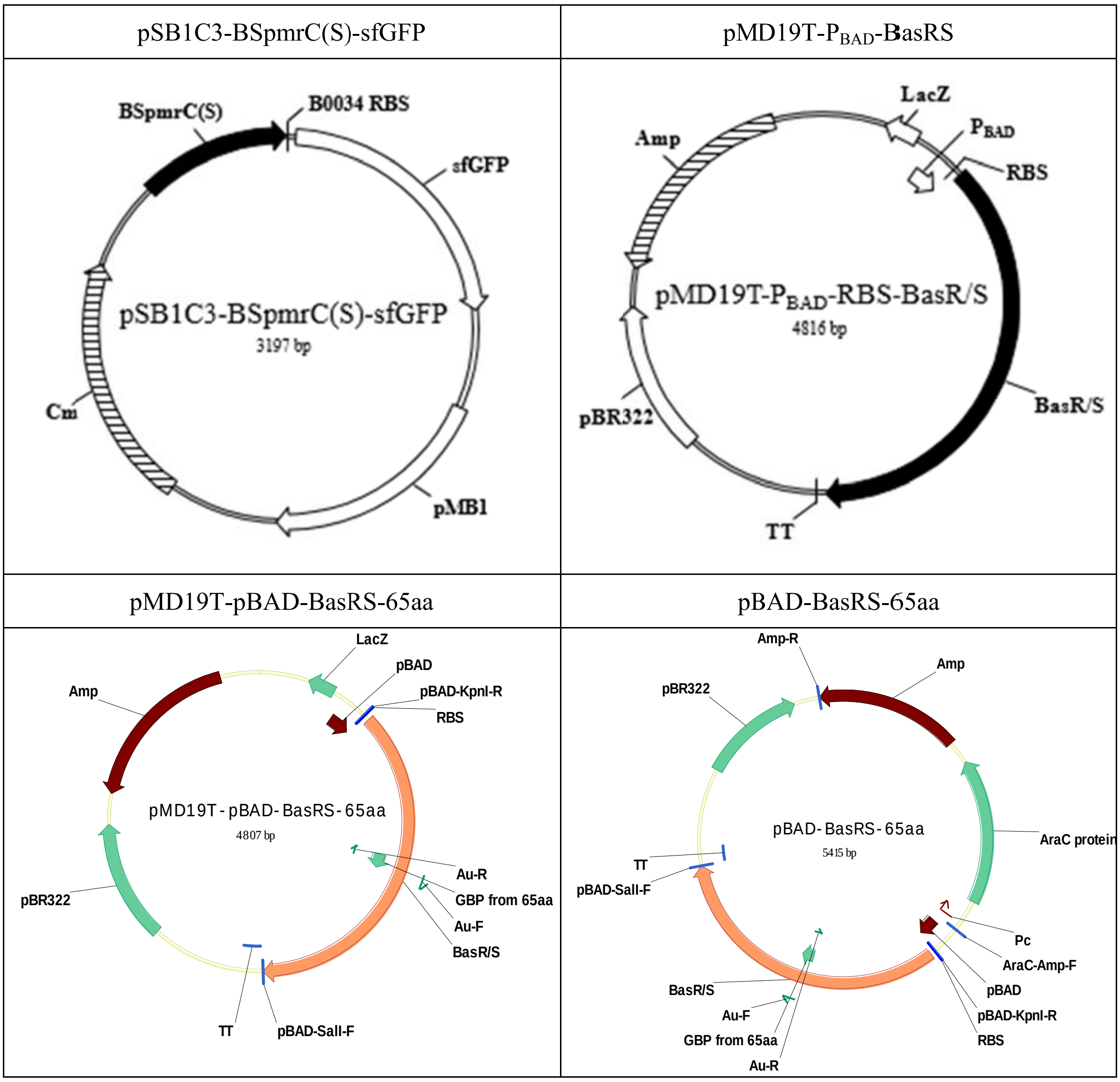

Fig. S1 The construction map of pSB1C3-BSpmrC(S)-sfGFP, pMD19T-P_{BAD}-BasRS, pMD19T-pBAD-BasRS-65aa and pBAD-BasRS-65aa.

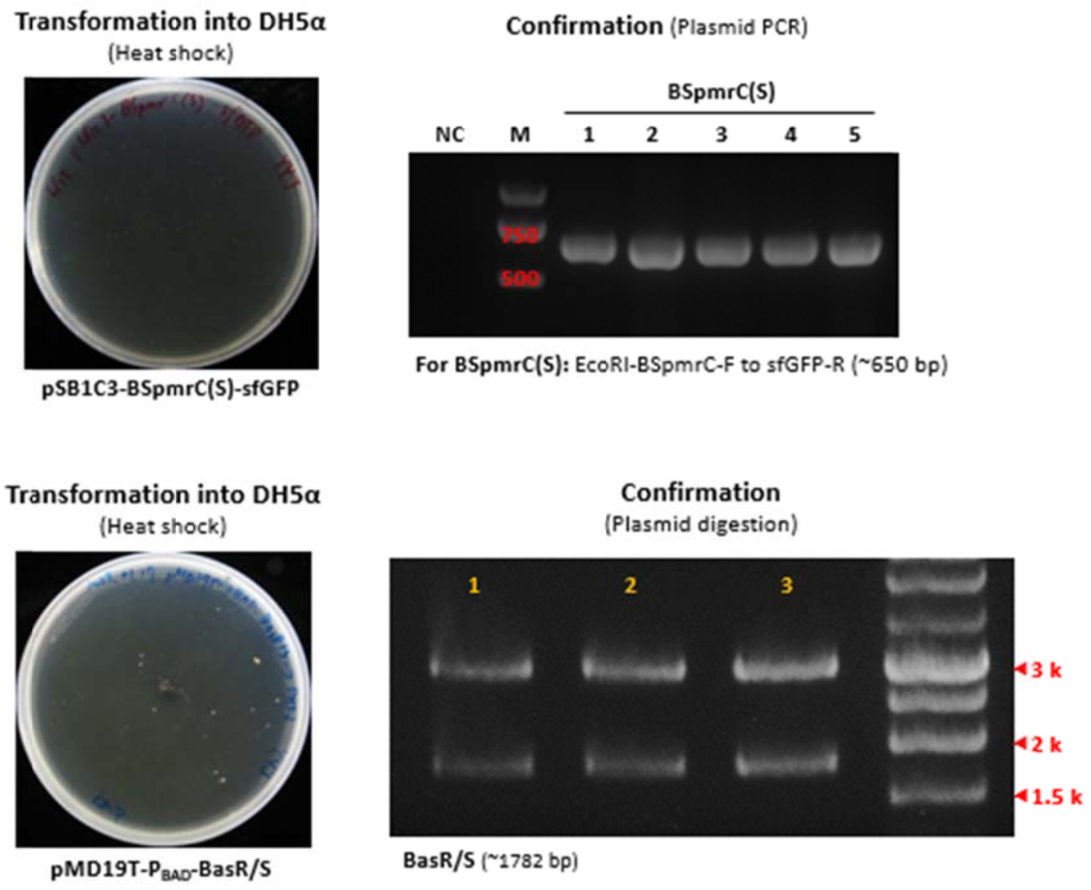

Fig. S2 The transformation and confirmation process of pSB1C3-BSpmrC(S)-sfGFP (upper) and pMD19T-P_{BAD}-BasR/S (lower).

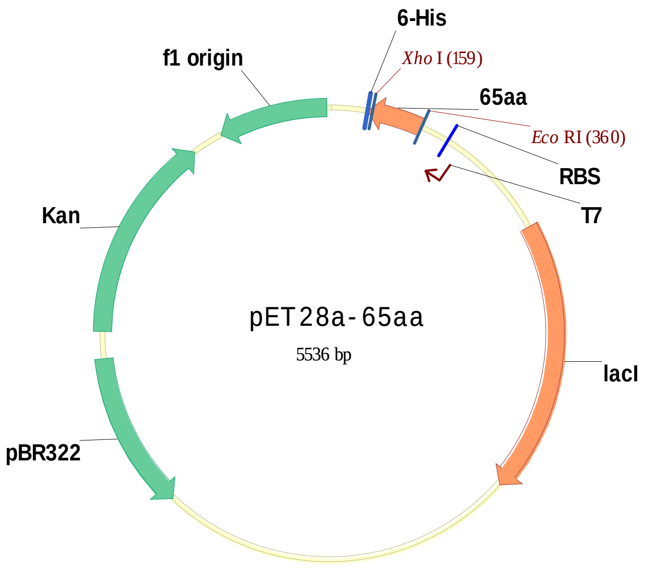

Fig. S3 The construction map of pET28a-65aa.

Table S1 All represented *E. coli* strains with PmrA/PmrB two-component system

Material	Description
pMD19T-P_{BAD}-BasRS/DH5α	DH5α strain contains BasRS plasmid
pSB1C3-BSpmrC(S)-sfGFP/DH5α	DH5α strain contains BSpmrC(S)-sfGFP plasmid
MG1655ΔBasS::Kan	MG1655 mutant strain, replaced *basS* in chromosome with Kan
pMD19T-P_{BAD}-BasRS/MG1655ΔBasS::Kan	MG1655 mutant strain contains BasRS plasmid
pMD19T-P_{BAD}-BasRS+pSB1C3-BSpmrC(S)-sfGFP /MG1655ΔBasS::Kan	MG1655 mutant strain contains BasRS and BSpmrC(S)-sfGFP plasmids for sensing iron ion.
pMD19T-pBAD-BasRS-65aa +pSB1C3-BSpmrC(S)-sfGFP /MG1655ΔBasS::Kan	MG1655 mutant strain contains BasRS-65aa and BSpmrC(S)-sfGFP plasmids for sensing gold ion.
pBAD-BasRS-65aa +pSB1C3-BSpmrC(S)-sfGFP /MG1655ΔBasS::Kan	MG1655 mutant strain contains BasRS-65aa under araC regulation and BSpmrC(S)-sfGFP plasmids for improvement of sensing gold ion.

Table S2. The amino acid and DNA sequence of 65aa.

Amino acid sequence	MQHVFTVDGMSCGHCVKAITQAIRALDPQAQVRVDLDERRVEVESDRSRVALA<br>DAIRDEGYTVRD
DNA sequence	ATGCAGCATGTGTTTACCGTGGATGGCATGAGCTGCGGCCATTGCGTGAAAG<br>CGATTACCCAGGCGATTCGCGCGCTGGATCCGCAGGCGCAGGTGCGCGTGGA<br>TCTGGATGAACGCCGCGTGGAAGTGGAAAGCGATCGCAGCCGCGTGGCGCT<br>GGCGGATGCGATTCGCGATGAAGGCTATACCGTGC GCGAT
